# Diagnostic yield of proximal jejunal lesions with third‐generation capsule endoscopy

**DOI:** 10.1002/deo2.134

**Published:** 2022-06-12

**Authors:** Issei Hirata, Akiyoshi Tsuboi, Shiro Oka, Akihiko Sumioka, Sumio Iio, Yuichi Hiyama, Takahiro Kotachi, Ryo Yuge, Ryohei Hayashi, Yuji Urabe, Shinji Tanaka

**Affiliations:** ^1^ Department of Gastroenterology and Metabolism Hiroshima University Hospital Hiroshima Japan; ^2^ Department of Endoscopy Hiroshima University Hospital Hiroshima Japan; ^3^ Department of Center for Integrated Medical Research Hiroshima University Hospital Hiroshima Japan; ^4^ Division of Regeneration and Medicine Center for Translational and Clinical Research Hiroshima University Hospital Hiroshima Japan

**Keywords:** capsule endoscopy, diagnostic yield, double‐balloon endoscopy, proximal jejunum, small‐bowel lesions

## Abstract

**Objectives:**

Capsule endoscopy (CE) has been shown to have poor diagnostic performance when the capsule passes quickly through the small bowel, especially the proximal jejunum. This study aimed to evaluate the diagnostic yield of proximal jejunal lesions with third‐generation CE technology.

**Methods:**

We retrospectively examined 138 consecutive patients, 76 (55.0%) of whom were men. The patients’ median age was 70 years, and proximal jejunal lesions were detected by CE and/or double‐balloon endoscopy at Hiroshima University Hospital between January 2011 and June 2021. We analyzed the diagnostic accuracy of CE for proximal jejunal lesions and compared the characteristics of the discrepancy between the use of CE and double‐balloon endoscopy with Pillcam SB 2 (SB2) and Pillcam SB 3 (SB3).

**Results:**

SB2 and SB3 were used in 48 (35%) and 90 (65%) patients, respectively. There was no difference in baseline characteristics between these groups. Small‐bowel lesions in the proximal jejunum comprised 75 tumors (54%), 50 vascular lesions (36%), and 13 inflammatory lesions (9%). The diagnostic rate was significantly higher in the SB3 group than in the SB2 group for tumors (91% vs. 72%, *p* < 0.05) and vascular lesions (97% vs. 69%, *p* < 0.01). For vascular lesions, in particular, the diagnostic rate of angioectasia improved in the SB3 group (100%) compared with that in the SB2 group (69%).

**Conclusions:**

SB3 use improved the detection of proximal jejunal tumors and vascular lesions compared with SB2 use.

## INTRODUCTION

Recently, advances in technology have made it possible to visualize the entire small bowel using endoscopic systems, such as capsule endoscopy (CE)^1^ and balloon endoscopy.^2^ The indications for small‐bowel endoscopy include obscure gastrointestinal bleeding, small‐bowel stenosis, tumors, and inflammatory bowel disease.^3^, ^4^, ^5^, ^6^, ^7^, ^8^, ^9^ Previous studies have shown that the diagnostic yield of CE and balloon endoscopy in patients with the suspected small‐bowel disease is comparable.^10^, ^11^, ^12^ However, with CE, a significant number of lesions are missed because images are either not captured (in cases when the capsule endoscope runs out of the battery or a lesion is located in the blind loop) or are of poor quality.^13^, ^14^ Poor‐quality images are obtained when the capsule passes quickly through the proximal jejunum^15^ and lesions in the proximal jejunum tend to be missed as a result.^12^, ^15^ Bubble artifacts and relatively poor luminal distension sometimes influence CE readings.

The Pillcam SB3 (SB3) video capsule (Covidien, Mansfield, MA, USA), a third‐generation CE, has a larger field of view than the Pillcam SB2 (SB2) and automatically adjusts the imaging frame rate according to the speed of the capsule's passage through the small bowel. This adaptive frame rate (AFR) feature of SB3 relies on the communication between the DR3 PillCam recorder. While the frame rate of SB2 is fixed at two images per second, SB3 can receive six images per second depending on the speed of the capsule.^16^, ^17^ Although there have been some reports comparing the detection rate of duodenal papillae between SB3 and older generation devices,^18^, ^19^, ^20^ there are no reports on the diagnostic yield of SB3 for lesions in the proximal jejunum.

Thus, we aimed to evaluate the diagnostic yield of proximal jejunal lesions using SB3 and compare it to that using SB2, and evaluate the clinical characteristics of oversight small‐bowel lesions by CE.

## METHODS

### Patients

**FIGURE 1:**
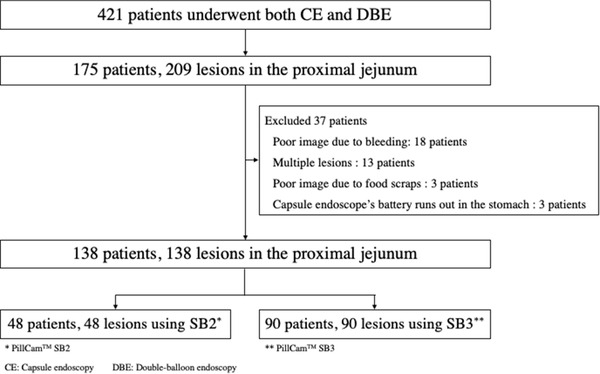
Flowchart of this study

We retrospectively examined 421 consecutive patients who underwent both CE and double‐balloon endoscopy (DBE) at Hiroshima University Hospital between January 2011 and June 2021. A flowchart of the enrolled patients is presented in Figure 1. A total of 175 patients with 209 lesions—which were identified in the proximal jejunum using DBE—were included in this study. We excluded those with poor images or multiple lesions, and those in whose stomachs the capsule endoscope battery ran down. Finally, 138 patients with a total of 138 lesions were enrolled in this study; 48 and 90 underwent CE using SB2 and SB3, respectively. CE was performed using SB2 devices from January 2011 to March 2014 and SB3 devices after April 2014.

This study was performed in accordance with the principles of the Declaration of Helsinki. All patients were informed of the risks and benefits of CE and DBE, and each patient provided written informed consent for the procedure to be performed. No patient declined participation during the study period. This study was approved by the Institutional Review Board at Hiroshima University Hospital (registration number: E‐1143).

### CE procedure

CE was performed using SB2 or SB3 devices. The capsule was swallowed with a solution of dimethicone after an overnight fast without any other preparation. Patients were allowed to drink clear liquids for 2 h and eat light meals for 4 h after swallowing the capsule. After 8 h, the sensor array and recording device were removed, and the images obtained were analyzed using Rapid Reader 6.5 or RAPID 8 workstation (Covidien). The capsule recordings were reviewed by two experienced physicians, each having read more than 100 capsule videos. The final diagnosis was made by two endoscopists with reference to the CE findings. In cases of disagreement, a diagnostic consensus was reached through discussion.

### DBE procedure

DBE was performed using EN‐450P5/20, EN‐450T5/W, EI‐530B, EI‐580BT, or EN‐580T (FUJIFILM Medical Co., Ltd., Tokyo, Japan). Antegrade DBE was performed after an overnight fast. Within 1 week of CE, DBE was performed by two experienced endoscopists. Patients were sedated with midazolam, pethidine, or pentazocine, if necessary. Blood pressure, heart rate, and oxygen saturation were monitored during DBE, and biopsy specimens were obtained when needed. The final diagnosis was made in the same way as that following CE.

### Evaluation

We evaluated the following: the breakdown of lesions detected by DBE, the diagnostic concordance rate of CE and DBE, and the characteristics of CE false‐negative lesions. The final diagnosis was made after histological examination of endoscopically or surgically acquired biopsy specimens to determine the accuracy of the CE and DBE findings. In this study, the proximal jejunum was defined as an area up to 50 cm from the ligament of Treitz on fluoroscopy during DBE.

### Statistical analysis

Continuous data are reported as means ± standard deviations and ranges, and non‐normally distributed data are reported as median and inter‐quartile range (IQR). Comparisons were performed using the chi‐square test, Fisher's exact test, and Mann–Whitney *U* test for categorical data. Statistical significance was set at a *p*‐value of < 0.05. The software program JMP Pro 15 (SAS, Cary, NC, USA) was used for statistical analyses.

## RESULTS

The clinical characteristics of the enrolled patients are presented in Table 1. Seventy‐six (55%) of the 138 patients enrolled in this study were men. The median age of the patients was 70 years (IQR, 61–77 years), and there were no differences in baseline characteristics between the SB2 and SB3 groups. The most frequent indication for CE was obscure gastrointestinal bleeding (82 patients, 59%), which was overt in 63 patients (45%) and occult in 19 (14%).

**TABLE 1:** Characteristics of the enrolled patients

**Variables**	**Capsule endoscopy**		
	**SB2, *n* = 48**	**SB3, *n* = 90**	***p*‐value**
Sex (male, number,%)	28 (58)	48 (53)	0.57
Age (years), median (IQR)	72 (62–81)	70 (60–81)	0.32
Body mass index (mean ± SD)	21.5 ± 3.4	21.9 ± 3.9	0.52
Reasons for capsule endoscopy			0.10
Obscure gastrointestinal bleeding	29 (60)	53 (60)	
Overt	24 (50)	39 (43)	
Occult	5 (10)	14 (16)	
Other image abnormality	8 (17)	25 (28)	
Abdominal symptoms	4 ( 8)	8 ( 9)	
Others	7 (15)	4 ( 4)	

Data expressed as the number (%). SB2, PillCam SB2; SB3, PillCam SB3; IQR, inter‐quartile range; SD, standard deviation.

The final diagnoses of the lesions are shown in Table 2 and were as follows: 75 tumors (54%), 50 vascular lesions (36%), and 13 inflammatory lesions (9%). The most frequent tumor in the proximal jejunum was malignant lymphoma (29 lesions, 21%), followed by an adenoma (11 lesions, 8%), gastrointestinal stromal tumor (GIST; nine lesions, 7%), and adenocarcinoma (nine lesions, 7%). Angioectasia (36 lesions, 26%) was the most common vascular lesion in the proximal jejunum. There were no significant differences in the final diagnoses between the two groups.

**TABLE 2:** Enrolled patients’ final diagnosis in the proximal jejunum

		**Capsule endoscopy**		
**Variables**	**Total**	**SB2, *n* = 48**	**SB3, *n* = 90**	***p*‐value**
Tumor	75 (54)	29 (60)	46 (51)	0.37
Malignant lymphoma	29 (21)	7 (15)	22 (24)	
Adenoma	11 (8)	4 (8)	7 (8)	
Gastrointestinal stromal tumor	9 (7)	4 (8)	5 (6)	
Adenocarcinoma	9 (7)	2 (4)	7 (8)	
Hamartomatous polyp	4 (3)	3 (6)	1 (1)	
Others	12 (9)	9 (19)	3 (3)	
Vascular lesion	50 (36)	16 (33)	34 (38)	0.71
Angioectasia	36 (26)	13 (27)	22 (24)	
Hemangioma	10 (7)	2 (4)	8 (9)	
Others	5 (4)	1 (2)	4 (4)	
Inflammation	13 (9)	3 (20)	10 (11)	0.54
Erosion	9 (7)	1 (2)	8 (9)	
Ulcer	4 (3)	2 (4)	2 (2)	

Data presented as the number (%). SB2, PillCam SB2; SB3, PillCam SB3.

The diagnostic yield of CE for the lesions is shown in Table 3. The overall detection rate for the various lesions in the proximal jejunum was significantly higher in the SB3 group than in the SB2 group (*p* = 0.001). For the tumor lesions, the detection rate of both proximal jejunal and vascular lesions was significantly higher in the SB3 group than in the SB2 group (*p *= 0.049 vs. *p = *0.009) In particular, the detection rate of angioectasia was significantly higher in the SB3 group than in the SB2 group (*p* = 0.0013). While the diagnosis rate of tumors represented by non‐submucosal tumors (SMT) was high with both SB2 and SB3 (95% and 100%), the diagnostic rate of tumors represented by SMT was higher in the SB3 group (79%) than in the SB2 group (42%, *p *= 0.06).

**TABLE 3:** Diagnostic yield of capsule endoscopy for lesions in the proximal jejunum

	**Capsule endoscopy**		
**Variables**	**SB2, *n* = 48**	**SB3, *n* = 90**	***P*‐value**
Tumor	72% (21/29)	91% (42/46)	0.049
SMT	42% (5/12)	79% (15/19)	0.056
Non‐SMT	94% (16/17)	100% (27/27)	0.386
Vascular lesion	69% (11/16)	97% (33/34)	0.009
Angioectasia	69% (9/13)	100% (22/22)	0.013
Hemangioma	50% (1/2)	88% (7/8)	0.377
Others	100% (1/1)	100% (4/4)	1.000
Inflammation	100% (3/3)	90% (9/10)	1.000
Erosion	100% (1/1)	88% (7/8)	1.000
Ulcer	100% (2/2)	100% (2/2)	1.000
Total	73% (35/48)	93% (84/90)	0.0010

SMT, submucosal tumor; SB2: PillCam SB2; SB3. PillCam SB3.

**FIGURE 2:**
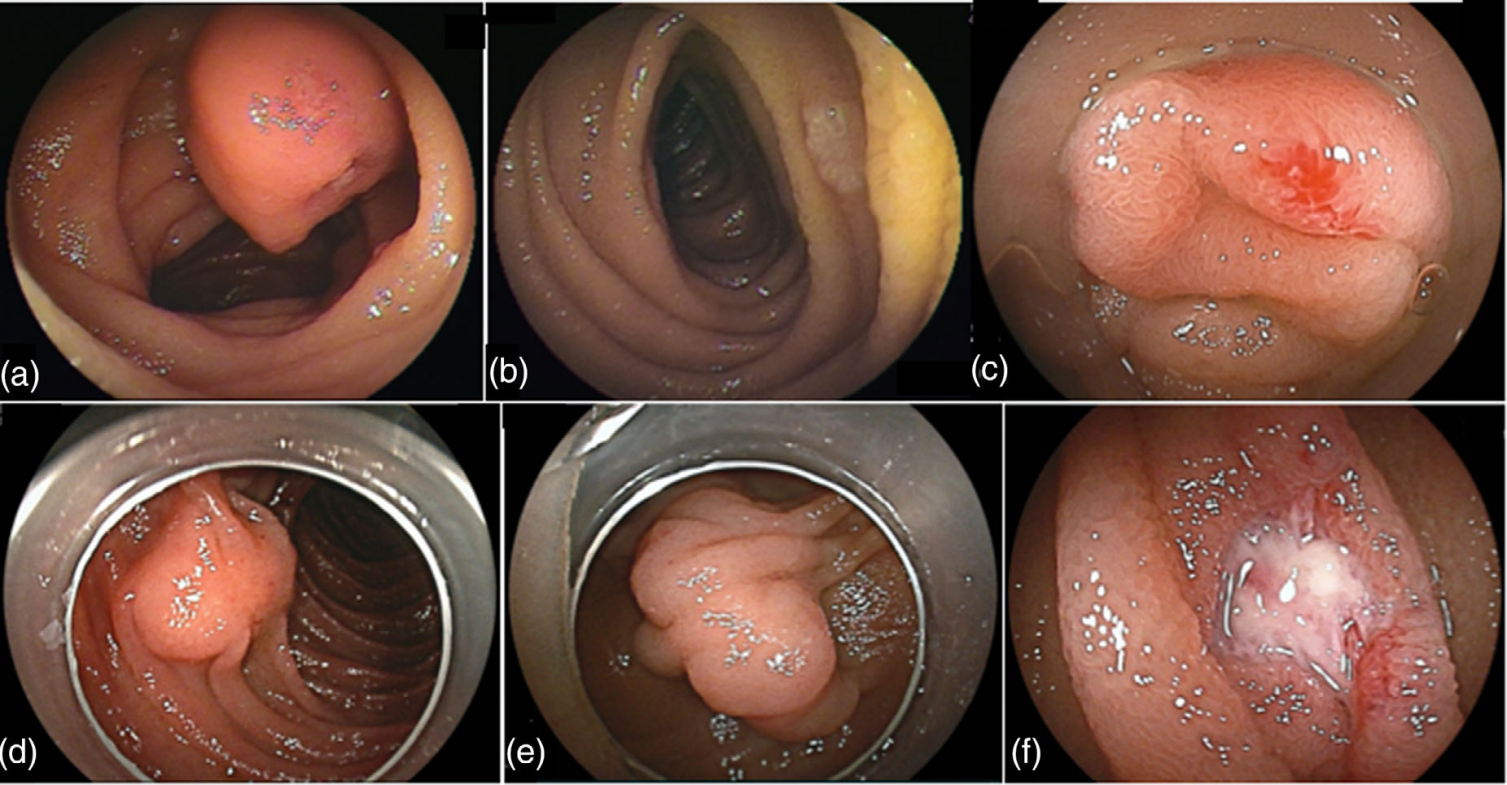
Capsule endoscopy with false‐negative lesions in the proximal jejunum. (a) Gastrointestinal stromal tumor (No. 2). (b) Adenoma (No. 9). (c) Angioectasia (No. 12). (d) Ectopic pancreatic tissue (No. 16). (e) Ileal duplication (No. 17). (f) Non‐specific ulcer (No. 19)

There were 19 cases of false‐negative CE, 13 of them were in the SB2 group, whereas six were in the SB3 group. The details of these cases are presented in Table 4. Among the false‐negative lesions, those larger than 20 mm were detected using contrast‐enhanced computed tomography (CT). Representative images of false‐negative lesions are shown in Figure 2. All lesions were SMTs or small lesions, such as those hidden behind the folds.

**TABLE 4:** False‐negative of capsule endoscopy in the proximal jejunum

**No**.	**Age (years)**	**Sex**	**Capsule endoscopy**	**Reason for capsule endoscopy**	**Diagnosis**	**Size of lesion (mm)**	**Form of lesion**
1	43	Male	SB2	Overt OGIB	GIST	50	SMT
2	81	Female	SB2	Overt OGIB	GIST	15	SMT
3	58	Female	SB2	Overt OGIB	Ectopic pancreatic tissue	30	SMT
4	55	Female	SB2	Overt OGIB	Ectopic pancreatic tissue	10	SMT
5	62	Male	SB2	Overt OGIB	Lipoma	20	SMT
6	36	Female	SB2	Other image of abnormalities	Lipoma	20	SMT
7	66	Female	SB2	Diarrhea	Ileal duplication	15	SMT
8	75	Male	SB2	Overt OGIB	Hemangioma	10	SMT
9	30	Male	SB2	Other image of abnormalities	Adenoma	2	Flat
10	66	Male	SB2	Overt OGIB	Angioectasia (Type 1b)	2	Red spot
11	61	Male	SB2	Overt OGIB	Angioectasia (Type 1b)	2	Red spot
12	87	Male	SB2	Overt OGIB	Angioectasia (Type 1a)	1	Red spot
13	84	Male	SB2	Overt OGIB	Angioectasia (Type 1a)	1	Red spot
14	44	Male	SB3	Other image of abnormalities	GIST	50	SMT
15	63	Male	SB3	Overt OGIB	GIST	20	SMT
16	70	Male	SB3	Overt OGIB	Ectopic pancreatic tissue	12	SMT
17	66	Male	SB3	Other image of abnormalities	Ileal duplication	20	SMT
18	74	Female	SB3	Overt OGIB	Hemangioma	10	SMT
19	77	Male	SB3	Occult OGIB	Non‐specific ulcer	5	Ulcer

GIST, gastrointestinal stromal tumor; OGIB, obscure gastrointestinal bleeding.

## DISCUSSION

Our study revealed that SB3 is a high‐quality modality with a high diagnostic yield for proximal jejunal lesions. Although the risk of oversight lesions has been reported for proximal jejunal lesions in previous reports,^12^, ^15^ to the best of our knowledge, there is no report on the diagnostic yield of SB3 for such lesions. In Japan, CE is considered the first‐line modality for small‐bowel diseases.^21^ Our findings showed a high diagnostic rate when SB3 was used, and the results are considered to endorse the clinical positioning of SB3.

Recently, several reports have demonstrated that a significant number (approximately 20%) of tumors in the small bowel can be missed with CE.^13^, ^22^, ^23^, ^24^, ^25^ The probability of false‐negative results in the detection of small‐bowel lesions in the proximal jejunum and ileum using SB2 is significant with CE.^4^ Nakamura et al.^26^ reported that SB2 could detect the esophageal‐cardiac junction, pyloric ring seen from the duodenal bulb, major papilla of the duodenum, ileocecal valve seen from the cecum, vermiform appendix, and anal canal in 17%, 33%, 18%, 20%, 3%, and 2% of cases, respectively. They concluded that SB2 is difficult to detect in areas of the gastrointestinal tract where the transit time of SB2 is short. SB3 has a significantly higher diagnostic yield for esophageal varices than SB2.^17^ SMTs are more likely to be missed with CE.^27^ In this study, the diagnostic yield of non‐SMT‐type tumors was 100%, while that of SMT‐type tumors was 79% using SB3. Of the five cases of false‐negative CE under SB3 with SMT‐type tumors, tumors larger than 20 mm in diameter (60%) were also detectable by CT in three and by DBE alone in two. Currently, CE is performed as a diagnostic inspection for obscure gastrointestinal bleeding,^21^, ^28^ and it is up to the attending physician to decide whether to perform DBE or follow up with the patient in the case of a negative CE result. It is better to perform oral DBE if possible because lesions in the proximal jejunum may be missed with CE.

**FIGURE 3:**
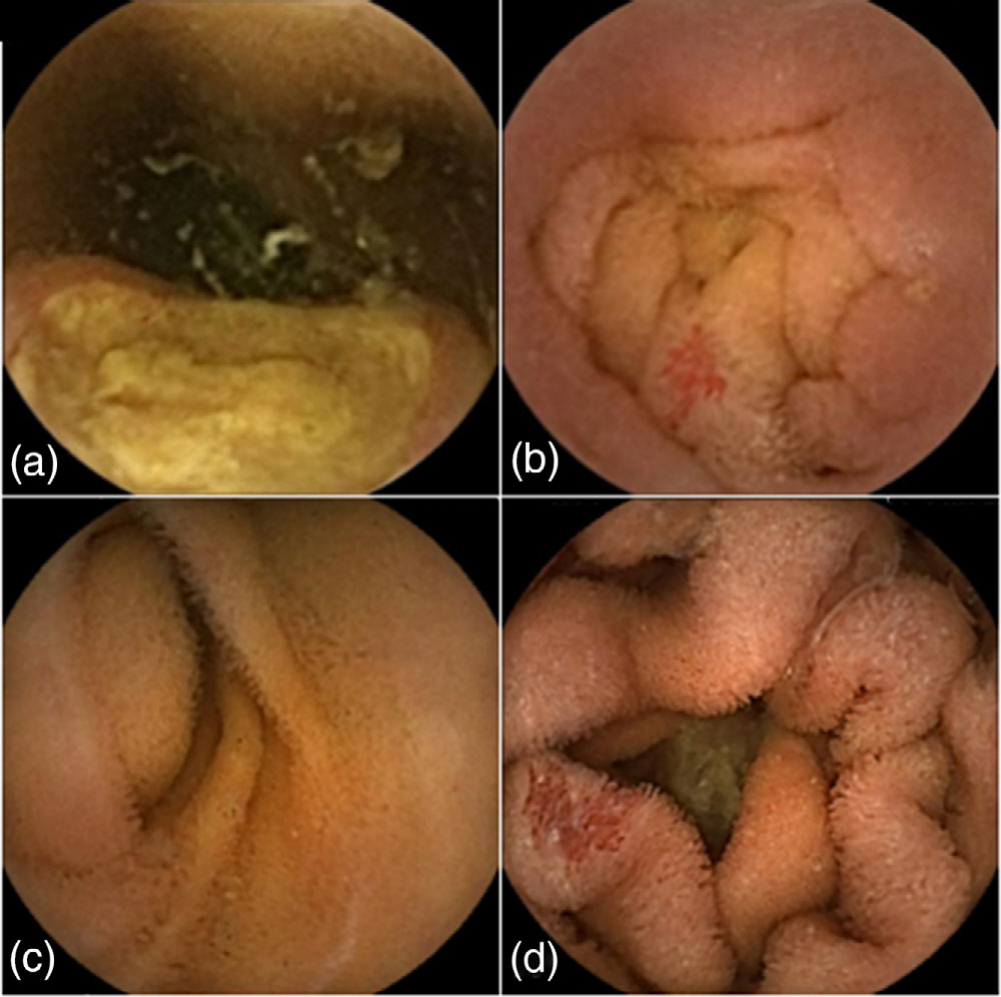
Capsule endoscopy images of proximal jejunal lesions. (a) Gastrointestinal stromal tumor (SB2). (b) Angioectasia (SB2). (c) Gastrointestinal stromal tumor (SB3). (d) Angioectasia (SB3)

SB3 also offers superior image quality compared with SB2. The minimum detectable object size for SB3 (0.07 mm) is shorter than that for SB2 (0.1 mm).^16^ The detection rate of angioectasia was the highest in SB3. This result is most likely because of the increased number of images captured with AFR (SB2, 2 frames per second; SB3, 2 or 6 frames per second); however, it is possible that the improved image quality enhanced the visibility of small lesions, such as angioectasia. CE images of this study are presented in Figure 3. The usefulness of flexible spectral imaging color enhancement (FICE) in detecting small‐bowel lesions, such as angioectasia and erosion/ulceration in SB2, has been reported.^29^, ^30^ The combined use of FICE in SB3 may have further improved the diagnostic accuracy.

Recently, the field of artificial intelligence, also known as deep learning, has opened the door to more detailed image analysis and real‐time applications by automatically extracting relevant imaging features. Artificial intelligence‐assisted CE diagnosis has been reported and is suggested to be sensitive and specific enough to be introduced into clinical practice, where it will reduce the risk of missing lesions in the process of reading by physicians and potentially reduce the workload of physicians significantly.^31^, ^32^, ^33^, ^34^

In recent years, in addition to axial view CE, lateral/panoramic view CE has been used.^35^ Although the detection rate of the ampulla of Vater was reported at 18%–43% using Pillcam,^19^, ^25^ Friedrich et al. reported it to be 71% using panoramic view CE.^36^ In the duodenum, where the capsule has a faster transit speed, the detection rate of the ampulla of Vater is higher than that in conventional CE; this may be useful for detecting proximal jejunal lesions. The combined use of these modalities may further improve the detection rate of proximal jejunal lesions.

This study has some limitations. First, this was a single‐center, retrospective study. Second, the number of patients included was relatively small. Third, only lesions that could be diagnosed by DBE were studied, and cases missed by DBE were excluded. Thus, this study had a significant bias in patient selection because of the exclusion criteria. Fourth, we evaluated only proximal jejunal lesions and excluded those located in other parts of the small bowel. Thus, total enteroscopy was not achieved in all cases, and ileal cases were excluded from this study.

In conclusion, SB3 is an effective diagnostic modality for proximal jejunal lesions. However, there is still a risk of SMT oversight. Therefore, we think that further modality improvement (e.g., increased frame rate, improved viewing angle, and better image quality), development of maneuverable CE, and usefulness of capsules with a panoramic view should be considered in future research. Antegrade DBE should be performed even if there are no findings on CE in patients with the suspected small‐bowel disease.

## CONFLICT OF INTERESTS

The authors declare that they have no conflict of interest.

## FUNDING INFORMATION

None.
